# Exposure-dependent variation in cryolite induced lethality in the non-target insect, *Drosophila melanogaster*

**DOI:** 10.2478/intox-2014-0003

**Published:** 2014-07-16

**Authors:** Sayanti Podder, Sumedha Roy

**Affiliations:** Cytogenetics Laboratory, Department of Zoology, University of Burdwan, West Bengal, India

**Keywords:** cryolite, *Drosophila melanogaster*, fluoride, insecticide, LC_50_

## Abstract

The starting point of toxicity testing of any chemical in an organism is the determination of its Lethal Concentration 50 (LC_50_). In the present study, LC_50_ of a fluorinated insecticide cryolite is determined in a non-target insect model, *Drosophila melanogaster*. Interestingly, the result shows that acute LC_50_ of cryolite was much greater in comparison to the chronic one in case of *Drosophila* larvae. Larvae which were exposed to 65,000 to 70,000 µg/ml cryolite through food showed 50% mortality after 18 hours of acute exposure, whereas only 150 to 160 µg/ml cryolite was sufficient to cause 50% mortality in case of chronic exposure. Thus cryolite in a small amount when applied once cannot produce noticeable changes in *Drosophila*, whereas the same amount when used continuously can be fatal. The non-feeding pupal stage was also seen to be affected by chemical treatment. This suggests that the test chemical affects the developmental fate and results in failure of adult emergence. Absence of chemical-induced mortality in adults assumes that the toxicity of cryolite might be restricted to the preimaginal stages of the organism. Reduction in body size of larvae after ingestion of cryolite (with food) in acute treatment schedule is another interesting finding of this study. Some individuals consuming cryolite containing food cannot survive whereas the few survivors manifest a significant growth retardation which might be due to a tendency of refusal in feeding. Hence the present findings provide a scope of assessment of risk of other similar non-target groups.

## Introduction

The familiar fruit fly *Drosophila melanogaster* (Diptera: Drosophilidae) has worldwide distribution in different fruit gardens. It is not considered a pest. But *D. melanogaster* becomes an innocent victim when any pesticide is used to abolish harmful organisms. Most of the pesticides are persistent in the environment and cause serious trouble to non-target living animals due to their presence in the food chain (Karatas and Bahceci, 2009). Sodium hexafluoroaluminate, known as cryolite, is an insecticide used on many fruits, vegetables and ornamental crops for protection against leaf eating pests (Delong, 1934; Lawrenz *et al.,* 1939; US-EPA, 1997). It appears as colorless, glassy, white-reddish to grey-black prismatic monoclinic crystals. Cryolite occurs in two forms, natural and synthetic. But according to Largent (1948) synthetic cryolite may be a little more toxic than the natural mineral and acts as a stomach poisoning agent (Delong, 1934). The fluoride content of cryolite is 53.30%. It has been demonstrated that on toxicological ground, cryolite behaves as free fluoride (US-EPA, 1996) and it leaves fluoride as toxic residue in and on fruits and vegetables (Fluoride detective, 2012). This fluoride ion inhibits a variety of enzymes that contain iron, calcium and magnesium (Ware & Whitacre, 2004) in animal body.

*D. melanogaster* is popularly used as a model to study the toxic potential of any chemical (Jatav *et al.,* 2011). Many works have been done using *D. melanogaster* in laboratory condition to reveal well-defined effects of various insecticides and pesticides on the life cycle, hatchability and emergence of the fly (Nazir *et al*., 2001; Nazir *et al*., 2003; Gupta *et al*., 2005, Das *et al*., 2010). Fluorinated compounds such as sodium fluoride showed adverse effect on reproductivity of silkworm, depending on the fluoride tolerance of the variety of silkworm (Chen, 2003). But there are only few reports of LC_50_ (Lethal Concentration 50) or LD_50_ (Lethal Dose 50) of cryolite in invertebrates.

Lethal Concentration 50 or LC_50_ is a standard measure of toxicity to determine how much of a substance is needed to kill half of a group of experimental organisms in a given time. Determination of LC_50_ of cryolite in *D. melanogaster* is essential for selection of the concentration of the chemical for further experiments.

## Methods

### Experimental model

*Drosophila melanogaster* strain Oregon R was maintained on SDM (Standard Drosophila Medium) containing agar, corn meal, sucrose and yeast at 22±1 °C in laboratory conditions. Addition of propionic acid, nepagin (*n*-methyl-p-hydroxy benzoate) with rectified spirit was needed as a line of defense against infections.

### Chronic LC_50_ determination

The experimental concentrations of cryolite were selected for the chronic study in accordance with the concentration range which is applied in the field (US-EPA, 1997). Different concentrations of cryolite (10, 20, 40, 60, 80, 100, 120, 140, 160, 180 and 200 µg/ml) were dissolved in distilled water and mixed with SDM. Twenty 1^st^ instar larvae were introduced in food phials containing different concentrations of cryolite. Triplicate sets of each treatment group along with control group (also as triplicate) were considered for the study.

The number of pupae formed were recorded to provid the data for larval mortality (failure in pupation). Similarly the adult emergence in each treatment category was recorded indicating pupal death (failure in emergence). Emergence of flies was recorded till the 25^th^ day (since maximum flies emerge within 20–25 days in laboratory conditions, Podder & Ray 2013).

### Acute LC_50_ Determination

Another set of experiment was arranged by preparing cryolite containing food where cryolite concentrations were 10,000 µg/ml, 25,000 µg/ml, 50,000 µg/ml, 75,000 µg/ml and 100,000 µg/ml. Twenty numbers of early 3^rd^ instar larvae and twenty numbers of adult *D. melanogaster* were introduced in different food phials separately containing different concentrations of cryolite. Triplicate sets of each treatment group along with control group were also observed for the study. For determination of acute LC_50_, lethality of larvae and adults were recorded after 24, 48 and 72 hours.

The Lethal Dose 50 (LD_50_) of cryolite was reported 48,000 µg/ml in *D. melanogaster* (Mitchell and Gerdes, 1973). US-EPA (1997) reported acute oral LC_50_ of this chemical in rat to be greater than 50,000 µg/ml. Taking these as references, the acute LC_50_ study was carried out.

To have a clearer idea of the acute toxicity of the chemical, early third instar larvae were exposed to 3, 6, 9, 12, 15, 18, 21 and 24 hours to that specific concentration of the chemical which causes 50% larval mortality after 24 hours. Triplicate sets of the experiment were arranged taking twenty numbers of early 3^rd^ instar larvae in each and death of larvae was recorded after 3, 6, 9, 12, 15, 18, 21 and 24 hours.

### Statistical analysis of data

Probit analysis of the data has been done for the determination of LC_50_ of cryolite in 3rd instar larvae of *D. melanogaster.*

## Results

**Figure 1:**
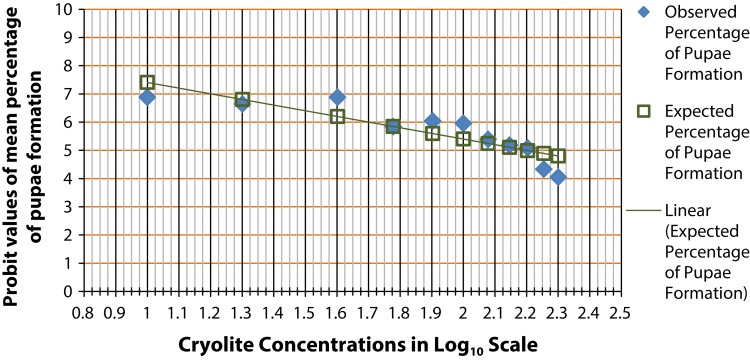
Probit analysis for chronic LC_50_ determination in larvae of *Drosophila melanogaster.* The graph shows the probit values of observed and expected percentage of pupae formation. Each treatment group comprises twenty 1^st^ instar larvae. Triplicate sets of such treatment groups along with control group were observed for the experimental duration of 25 days. The scattered dots representing the expected percentage of pupae formation are joined to form a trend line which help determine the log concentration of the chemical corresponding to 50% larval death (or 50% pupae formation). The LC_50_ of cryolite in log_10_ scale is between 2.175 to 2.2. The antilog value for the LC_50_ concentration is found to be in the range 150–158 µg/ml.

Figure 1 shows the chronic LC_50_ of cryolite in larvae of *D. melanogaster*, *i.e.*, 50% mortality in larval condition was observed due to intake of cryolite with food. Experimental insects receiving 10 and 40 µg/ml cryolite containing food showed equal percentage of pupae formation as in control group. In case of the 20 µg/ml treatment group, 95% pupae formation confirmed that the change in the number of pupae formation was negligible in comparison with control. Starting with 60 µg/ml, there was a decline in the percentage of pupae formation, which was reduced to 16.67% in case of 200 µg/ml treatment concentration. Thus, 50% larval mortality was recorded between 150 and 158 µg/ml treatment groups as presented in the graph.

**Figure 2:**
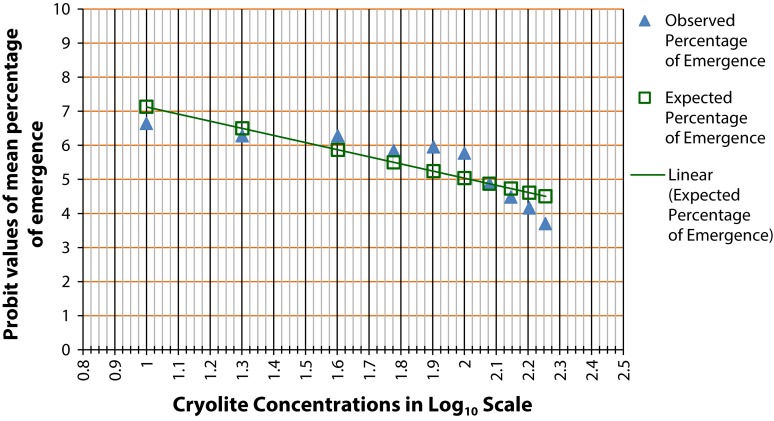
Probit analysis for chronic LC_50_ determination in pupae of *Drosophila melanogaster.* The graph shows the probit values of observed and expected percentage of pupae formation. Each treatment group comprises twenty 1^st^ instar larvae. Triplicate sets of such treatment groups along with control group were observed for the experimental duration of 25 days. The scattered dots representing the expected percentage of pupae formation are joined to form a trend line which help determine the log concentration of the chemical corresponding to 50% pupal death (or 50% emergence). The LC_50_ of cryolite in log_10_ scale is between 2 to 2.025. The antilog value for the LC_50_ concentration is found to be in the range 100–106 µg/ml.

Interestingly, Drosophilids manifested 50% pupal death at lower concentration of cryolite treatment (Figure 2) in comparison to LC_50_ of larvae. There is a similar decrease in the percentage emergence in treated individuals exposed to 60 µg/ml and the above treatment concentrations. The graph shows fluctuation in 10, 20 and 40 µg/ml treatment concentrations when compared with control. But 50% mortality of pupae was observed at 100 to 106 µg/ml treatment concentration.

**Figure 3:**
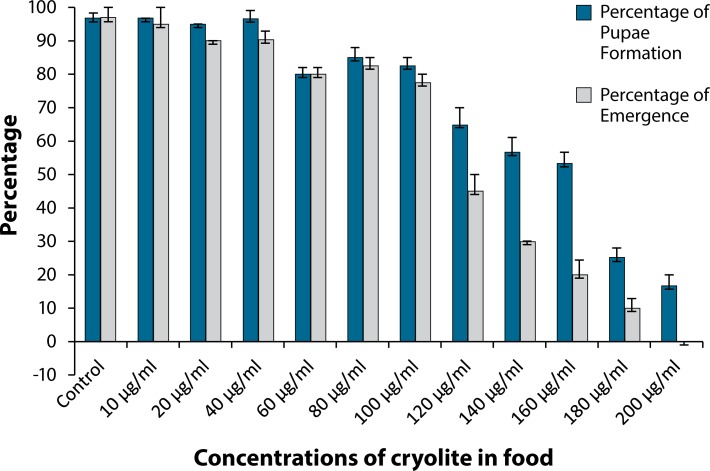
Comparison between percentage of pupae formation and percentage of emergence in *Drosophila melanogaster* at different concentrations of cryolite. Each column represents Mean Percentage ± Standard Error (SE) of three pooled determinations. Each pool consisted of 20 larvae. The vertical lines denote the Standard Error.

Figure 3 clearly depicts the distinct variation in response of developing larvae and developing pupae to different concentrations of cryolite. The chart shows the comparison between the percentage of pupae formation and emergence. Results indicate gradual decrease in percentages of both pupae formation and emergence starting with 80 µg/ml concentration of cryolite treatment. No fly came out from pupal case (no emergence) on treatment with 200 µg/ml cryolite.

For the second set of experiment, no lethality was found in case of adult *D. melanogaster* when placed in cryolite containing food (cryolite concentrations: 10,000 µg/ml to 100,000 µg/ml) for 24, 48 and 72 hours. But LC_50_ determination in case of larvae indicates different concentration ranges of cryolite at different time of exposure on acute basis.

**Figure 4:**
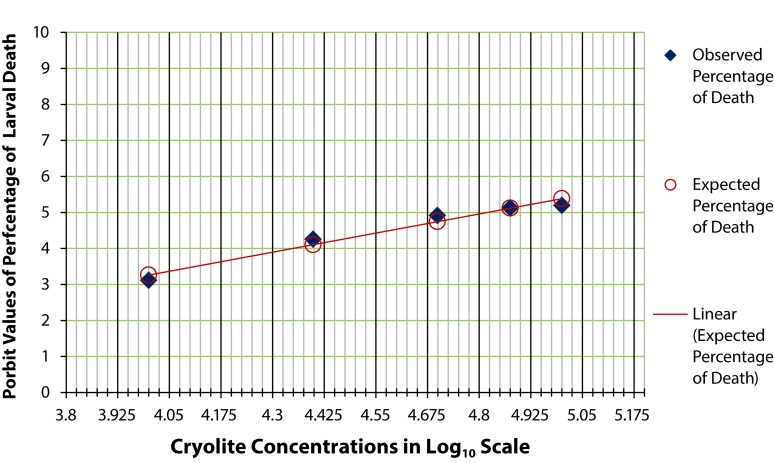
Probit analysis for acute LC_50_ determination of cryolite in larvae of *Drosophila melanogaster* after 24 hours. The graph shows the probit values of observed and expected percentage of larval death of three pooled determinations. Each pool consisted of 20 early 3^rd^ instar larvae. The scattered dots representing the expected percentage of larval death are joined to form a trend line which help determine the log concentration of the chemical corresponding to 50% larval death. The LC_50_ of cryolite in log_10_ scale is 4.825 after 24-hour exposure period. The antilog value for the LC_50_ concentration is 66,834.39; approximately 67,000 µg/ml.

**Figure 5:**
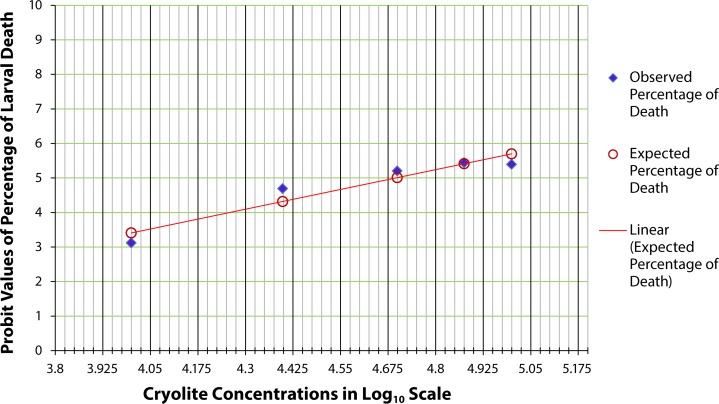
Probit analysis for acute LC_50_ determination of cryolite in larvae of *Drosophila melanogaster* after 48 hours. The graph shows the probit values of observed and expected percentage of larval death of three pooled determinations. Each pool consisted of 20 early 3^rd^ instar larvae. The scattered dots representing the expected percentage of larval death are joined to form a trend line which help determine the log concentration of the chemical corresponding to 50% larval death. The LC_50_ of cryolite in log_10_ scale is 4.7 after 48 hours exposure period. The antilog value for the LC_50_ concentration is 50,118.72; approximately 50,000 µg/ml.

**Figure 6:**
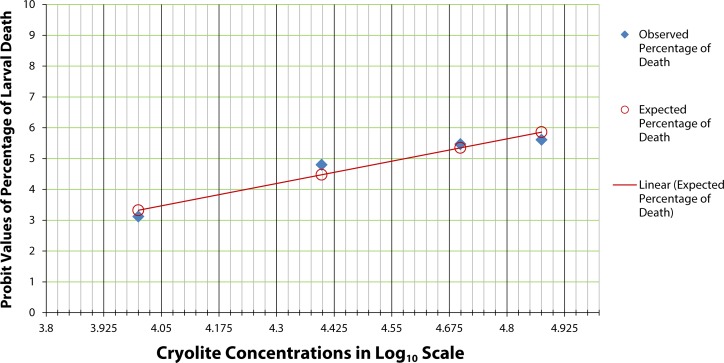
Probit analysis for acute LC_50_ determination of cryolite in larvae of *Drosophila melanogaster* after 72 hours. The graph shows the probit values of observed and expected percentage of larval death of three pooled determinations. Each pool consisted of 20 early 3^rd^ instar larvae. The scattered dots representing the expected percentage of larval death are joined to form a trend line which help determine the log concentration of the chemical corresponding to 50% larval death. The LC_50_ of cryolite in log_10_ scale is 4.575 after 48-hour exposure period. The antilog value for the LC_50_ concentration is 37,583.74; approximately 38,000 µg/ml.

**Figure 7:**
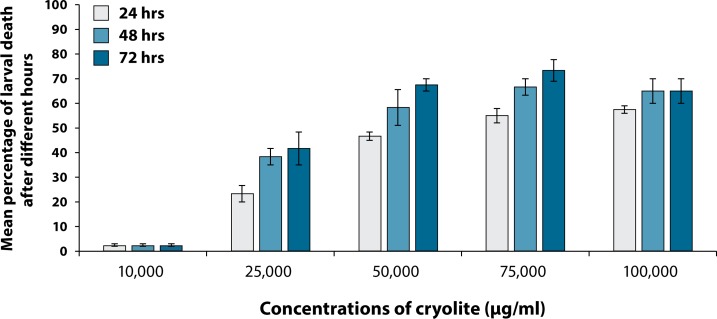
Comparison between percentages of larval death in *Drosophila melanogaster* at different concentrations of cryolite after acute exposures. Each column represents Mean Percentage ± Standard Error (SE) of three pooled determinations. Each pool consisted of 20 larvae. The vertical lines denote the Standard Error. Larval mortality is recorded after 24, 48 and 72 hours of chemical exposure.

At 24 hours, 50% larvae were found dead, when exposed to 67,000 µg/ml cryolite (Figure 4). But interestingly, after 48 hours, 50% mortality of the larvae was noted in 50,000 µg/ml concentration of treatment (Figure 5). Similarly was the effective LC_50_ concentration of cryolite 38,000 µg/ml for larvae exposed to treated food for 72 hours (Figure 6). Figure 7 represents the histogram showing differential percentages of larval death in *Drosophila* at three different exposure timings (24, 48 and 72 hours) of increasing concentrations of cryolite.

**Figure 8:**
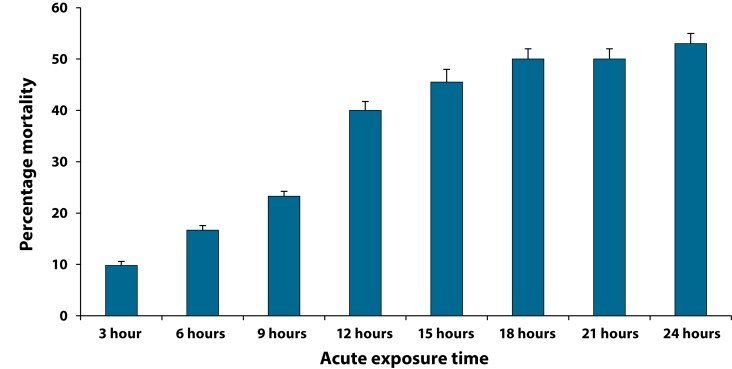
Percentage mortality of third instar larvae of *Drosophila melanogaster* subjected to differential exposure to LC_50_ of cryolite (67,000 µg/ml). Each column represents Mean Percentage ± Standard Error (SE) of three pooled determinations. Each pool consisted of 20 early 3^rd^ instar larvae. The vertical lines denote the Standard Error. Larval mortality is recorded at every 3-hour interval within 24 hours. Data thus collected help to decipher the actual time (18 hours) at which the lethal concentration causes 50% mortality.

On further splitting up of the 24 hour duration, (Figure 8) it was seen that 50% of larvae were dead by 18 hours of exposure to 67,000 µg/ml cryolite.

**Figure 9:**
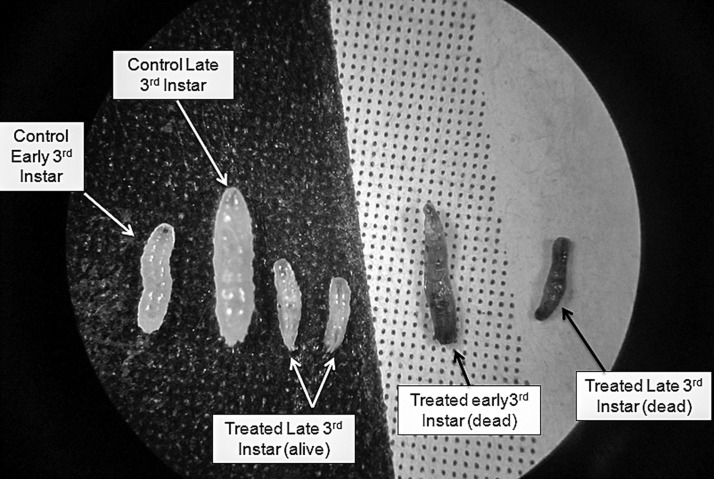
Treated early and late 3^rd^ instar larvae of *Drosophila* showing compressed body size. The reduced size indicates survival strategy adopted by larvae at different concentrations of acute cryolite exposure. Blackish larvae indicate death due to chemical intake. Control larvae are used for comparison

**Figure 10:**
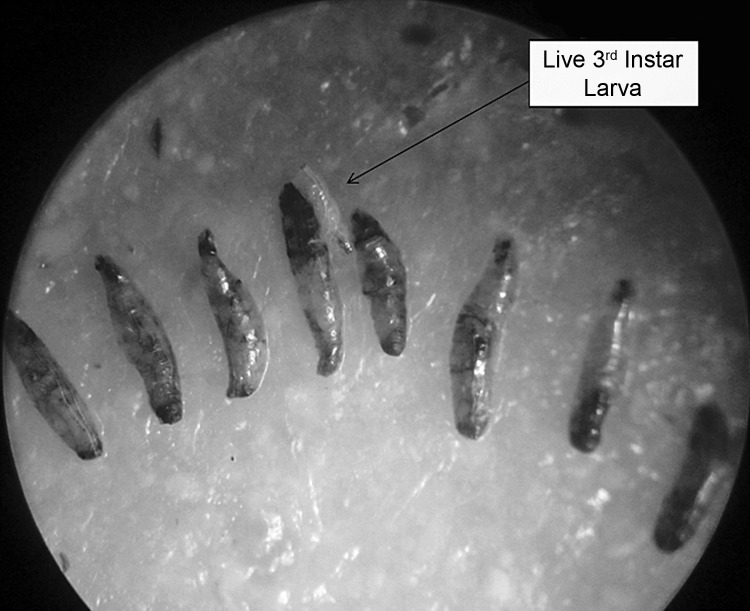
Dead early 3^rd^ instar larvae of *Drosophila* following cryolite exposure. Comparison with the single alive treated larva of the same age group, visible on the plate clearly indicates the size reduction resulting from chemical exposure.

Besides scoring for mortality of *D. melanogaster*, abnormalities in body size were noted among living individuals. The larvae which survived insults with higher concentrations of cryolite manifested reduction in size (Figure 9). Interestingly, the larvae that died during the first 24 hours of exposure showed blackening of the body. But the body size remained the same as during introduction within the treated food (Figure 10). The larvae that succumbed after 48 or 72 hours of chemical exposure were found to be much compressed in size and black in appearance (Figure 9).

It also seems to be noteworthy that a number of pupae failed to emerge from pupal case (Table 1) after chronic treatment with different concentrations of cryolite. The chemical concentration of 80 µg/ml and onwards showed gradual increase in percentage of pupal death which reached 100% in case of the 200 µg/ml treatment group.

**Table 1:** Mean percentage of pupal death.

Concentrations of Cryolite	Mean percentage
Control	0
10 µg/ml	1.73
20 µg/ml	5.26
40 µg/ml	6.49
60 µg/ml	0
80 µg/ml	2.94
100 µg/ml	6.06
120 µg/ml	30.77
140 µg/ml	47.06
160 µg/ml	62.5
180 µg/ml	60
200 µg/ml	100

## Discussion

Acute toxicity determines the toxic potential of any pesticide in any organism after a single short-term exposure. Acute toxicity is generally known to be based on the prompt effect of a chemical within a 24-hour exposure period. In the present study, 24-hour exposure to cryolite containing food medium at 67,000 µg/ml concentration killed 50% larvae of *D. melanogaster*. Hence the acute LC_50_ seems to be around these concentrations. But interestingly, chronic exposure to 150–158 µg/ml cryolite in SDM caused 50% larval mortality. The data for chronic LC_50_ recorded death caused by slow and delayed poisonous effects; hence this concentration range can be referred to as chronic LC_50,_ which is much lower in comparison with acute LC_50_ concentrations.

The toxic effect of any chemical following a single acute exposure may be quite different from the effects produced by chronic exposure. It was reported that a small amount of cryolite at one-time application is not sufficient to produce detectable changes in the biology of the animal, while the same small amount of the chemical applied day after day may cause chronic illness and ultimate death. At lower concentrations, insects try physiologically to combat the poisonous effects of any chemical by its elimination through the intestinal tract. According to Largent (1948), rapid elimination of cryolite from the rat digestive tract can save the animal from harmful effects. A similar response is also true for Drosophilids. As observed, beyond a certain threshold concentration (200 µg/ml), the *D. melanogaster* body fails the tussle and surrenders. This concentration of chronic treatment reveals the longest larval duration when compared to other treatment categories (Podder & Roy, 2013).

A lower concentration of any chemical might be more effective when applied for a longer duration. Hence exposure duration to any chemical is a very important and deciding factor in the process of LC_50_ determination.

The present study also revealed that the percentage of larval death increased with increase in treatment concentration, signifying that exposure concentration is yet another deciding factor. The larval death rate increased after increasing acute exposure with 25,000; 50,000; 75,000 µg/ml for 24, 48 and 72 hours but further increase in treatment to 100,000 µg/ml was found to be ineffective. This seems to be due to saturation after 48 h of exposure, which inhibits further increase in the percentage of death at 72 hours.

For any holometabolous insect, the different life stages are very important markers to evaluate any undesired effect. In the present study with *D. melanogaster,* only 50% adult emergence was noted with chronic treatment of cryolite at 100–106 µg/ml concentration. Emergence happens to be a powerful endpoint to detect life cycle anomalies in *Chironomus riparius* exposed to polycyclic aromatic compounds (Paumen *et al.,* 2008). Similarly, pupation and emergence are considered to be of the most sensitive indicators of copper toxicity in *Chironomus ramosus* by Majumdar and Gupta (2012). The most important and intriguing part of the pupal stage is that it is an essential metabolically active developmental stage devoid of feeding where maximum tissue rearrangements are taking place. Thus, food already ingested in the larval life happens to manifest its effect in pupae (Khan *et al*., 1991; Dad *et al*., 2011). In the present study cryolite (at some of the treatment concentrations) consumed through food in the larval life might be sub-lethal, hence larvae succeeded to pupate. Some pupae failed to attain adulthood, while others evaded pupal death and emerged successfully. The control pupae manifested 100% emergence, hence 0% pupal deaths. Similarly, 60 µg/ml treatment concentration caused 0% pupal death, but the rate of pupae formation in this case was seen to be affected by the treatment. Up to the 100 µg/ml concentration, the treatment caused nominal pupal death suggesting maximum response of the chemical restricted to the larval life, whereas concentrations beyond the range caused significant changes in the response pattern with distinct rise in pupal deaths. The fluoride ions of cryolite might inhibit a variety of enzymes that contain iron, calcium and magnesium (Ware & Whitacre, 2004) in experimental insect and thus be responsible for death and delay in life stages. Here no adult emergence was seen with 200 µg/ml treatment. This finding can well be corroborated with the findings of Jahan *et al.* (1990), who observed failure in emergence in *Musca domestica* after treatment with 0.03% Azadirachtin ^®^. But most interestingly, acute treatment in adult flies in the present treatment concentrations did not cause any significant mortality. Though the reason behind this remains uncertain yet it can be hypothesized that the adult flies took a much less amount of food, which might be the reason for this inert response.

The eggs of *D. melanogaster* under the given laboratory conditions hatched after 24 hours and the resulting larvae pupate within 9 days. According to Edgar and Orr-Weaver (2001), larvae enhance their body mass 200 fold by increase in cell size. This growth is stimulated by food intake which causes endocycles in larval body leading to DNA replication resulting in cell size enlargement but not cell division (Reddy *et al*., 2006). The study also showed reduction in larval size after acute treatments. Ellisor and Floyed (1939) found similar shrinkage in body size of *Ascia rapae* after insult through cryolite containing food and they hypothesized that larvae refused to feed on chemical contaminated food for several days. Similarly, Wene and Hansberry (1944) noted that many been beetle larvae did not ingest lethal amount of cryolite from food at a 24-hour period and the survivors manifested significant retardation in growth and development. The results of the present study equally depict growth retardation in larvae treated at concentrations which have successfully expressed developmental delay in one of our previous works (Podder & Roy, 2013).

Hence the study provides information regarding the lethal concentration 50 of the insecticide cryolite in an insect which is considered to be a non-target organism. Not only that, the results highlight the fact that long-term exposure to low concentrations of the test chemical is equally potent to elicit toxic response and be lethal when compared to acute treatments with higher concentrations of the chemical. Thus the present study suggests that irrational use of such insecticides should be minimized.
